# Rapid, sensitive, and visual detection of porcine rotavirus with RPA-CRISPR/Cas13

**DOI:** 10.3389/fmicb.2025.1617955

**Published:** 2025-08-20

**Authors:** Yong Mi, Mingyang Dai, Xiaokun Xing, Yang Zhang, Zhiwen Xu, Ling Zhu

**Affiliations:** ^1^College of Veterinary Medicine, Sichuan Agricultural University, Chengdu, China; ^2^Key Laboratory of Animal Disease and Human Health of Sichuan Province, Chengdu, China

**Keywords:** porcine rotavirus, RPA, CRISPR/Cas13, on-site detection, one-tube

## Abstract

Porcine rotavirus (PoRV) is one of the major pathogens causing viral enteritis in piglets, posing serious threats to the pig industry and public health. Existing pathogen detection methods, such as RT-qPCR, suffer from complex procedures and strong reliance on equipment, making them difficult to meet the needs of grassroots laboratories or field detection. Therefore, in this study, a novel rapid and visual detection platform, was developed based on the CRISPR/Cas13 system. This platform integrates Recombinase Polymerase Amplification (RPA), T7 transcription, CRISPR trans-cleavage, and fluorescence signal output. Targeting the PoRV VP6 gene, it achieves highly sensitive and specific detection of PoRV without the need for large equipment. The optimized system has a minimum detection limit of 10^1^ copies/μL for standard plasmids, and it was validated with 62 clinical diarrheal samples, yielding results consistent with RT-qPCR, demonstrating good clinical applicability. This platform is simple to operate, intuitive in detection, and cost-effective, making it suitable for rapid field screening of PoRV in resource-limited areas. It provides robust technical support for early diagnosis and epidemiological investigation of porcine rotavirus.

## 1 Introduction

Rotavirus (RV) is a leading cause of viral enteritis in infants, young children, and juvenile animals worldwide. RV is primarily transmitted via the fecal-oral route, with infected individuals typically exhibiting symptoms such as diarrhea, vomiting, and reduced appetite (Gao et al., 2023). This infection can be transmitted between humans and animals, representing a significant global public health concern. RV is classified into 10 groups (A-J) based on the antigenic relationships of its VP6 protein, with types I and J recently identified in shelter dogs in Hungary and bats in Serbia (Vlasova et al., 2017). The outer capsid proteins VP7 and VP4 induce neutralizing antibodies and form the basis of the G and P dual genotyping system (Papp et al., 2013). The most common groups infecting humans and animals are groups A, B, and C (RVA, RVB, and RVC), among which RVA has the highest prevalence and poses the greatest threat. To date, at least 42 G genotypes and 58 P genotypes have been identified in humans and animals (Ren et al., 2022). A total of twelve G genotypes (G1 to G6, G8 to G12, and G26) and sixteen P genotypes (P [1] to P [8], P [11], P [13], P [19], P [23], P [26], P [27], P [32], and P [34]) have been associated with pigs (Vlasova et al., 2017). RV has a profound impact on global public health and the swine industry. Given the absence of specific treatments for RV, early and accurate diagnostic methods are essential for its prevention and control.

Serological and pathogenetic detection are the primary methods for diagnosing RV. Culture-based isolation methods, particularly the isolation and identification of the virus using Vero or MA104 cells after the addition of trypsin, are considered the most direct and accurate approaches, thus establishing them as the gold standard for viral diagnosis (Richman et al., 1984). However, pathogen isolation involves meticulous procedures and extensive cell culture cycles, along with inherent uncertainties in the isolation process. Specifically, virus isolation often requires multiple attempts, as achieving success on the first try is rare. This unpredictability makes it challenging to rely on this method for large-scale pathogen screening or epidemiological studies. Molecular techniques, such as RT-PCR and RT-qPCR, offer sensitive and precise diagnostic capabilities but are overly dependent on complex procedures and specialized equipment, making them unsuitable for use in field or basic laboratory settings. Serological diagnostics, such as ELISA, are practical and easy to implement but are still prone to cross-reactivity and false positives. Considering that pig farms affected by RV are often located in complex field environments, remote villages, and resource-limited areas, there is an urgent need for a rapid, stable, portable, visual, and equipment-free detection platform to address the threat posed by RV.

Clustered Regularly Interspaced Short Palindromic Repeats (CRISPR) systems are a key component of the microbial adaptive immune system, responsible for recognizing and eliminating foreign nucleic acids based on their sequences (Paul and Montoya, 2020). The target recognition and nonspecific trans-cleavage activities of the CRISPR/Cas system have demonstrated great potential in the development of *in vitro* nucleic acid detection technologies. Various detection methods based on the CRISPR-Cas system have been described, such as one-Hour Low-cost Multipurpose highly Efficient System (HOLMES), DNA Endonuclease-Targeted CRISPR Trans Reporter (DETECTR), and Specific High-sensitivity Enzymatic Reporter UnLOCKing (SHERLOCK) (Kellner et al., 2019; Li et al., 2019; Li et al., 2020). CRISPR-Cas13a can detect the presence of RNA targets through CRISPR RNA (crRNA) and the collateral cleavage activity of Cas13a. The crRNA recognizes the RNA target and activates the cleavage of nearby nontarget reporter RNAs (Gootenberg et al., 2017). SHERLOCK, a molecular detection platform based on Cas13a, integrates recombinase polymerase amplification (RPA), T7 transcription to amplify DNA into RNA, and the collateral activity of CRISPR-Cas13a (Kellner et al., 2019). However, the large size of Cas13a and its strict specificity for certain protospacer adjacent motifs (PAMs) limit its integration into various diagnostic platforms. The discovery of Cas13d effectively addresses these issues. Unlike previous systems, the CRISPR/EsCas13d system does not require specific PAMs for CRISPR RNA (crRNA), thereby broadening its range of applications and simplifying crRNA design (Qiao et al., 2021). At 112.4 kDa, Cas13d is significantly smaller than Cas13a, facilitating its activity expression. Therefore, Cas13d holds promise for overcoming the limitations of Cas13a, providing new pathways for nucleic acid detection (Wei et al., 2023). Currently, CRISPR-Cas13d-based detection has been successfully applied to pseudorabies virus, Japanese encephalitis virus, and other molecules (Feng et al., 2023; You et al., 2024a; You et al., 2024b). In this study, an enhanced Cas13d-based detection method was developed by combining RPA, T7 transcription and the trans-cleavage activity of CRISPR/Cas13d. This method enables sensitive, specific, equipment-free, and visual detection targeting the PoRV VP6 gene.

## 2 Materials and methods

### 2.1 Virus

Positive samples for porcine rotavirus (PoRV), porcine circovirus II (PCV2), porcine reproductive and respiratory syndrome (PRRSV), pseudorabies virus (PRV), porcine parvovirus (PPV), classical swine fever virus (CSFV), porcine epidemic diarrhea virus (PEDV), and Japanese encephalitis virus (JEV) are all stored in our laboratory. These samples include various types, such as tissue, fecal, serum, and samples from aborted fetuses. After being identified as positive, all samples are stored at −80°C. RNA or DNA was extracted from these samples using the TIANamp Virus DNA/RNA Kit (Tiangen, Beijing, China). cDNA was synthesized using PrimeScript RT Master Mix (Takara, Beijing, China), and both cDNA and DNA were stored at −80°C for future experiments.

### 2.2 Construction of standard plasmid

To ensure the broad applicability and reliability of the detection method, the highly conserved VP6 gene was selected from the PoRV sequences of different strains for the construction of a standard plasmid. The sequence details can be found in Supplementary file 1. The RV-positive plasmid pMD18-RV-VP6 was then synthesized by Sangon Biotech Co., Ltd. (Shanghai, China) and validated by PCR. The target gene sequence can be found in Supplementary file 1, and primer details are provided in Table 1.

**Table 1:** Nucleotide sequences.

Assay	Name	Sequences (5′–3′)
crRNA	crRNA1	CACCCGUGCAAAAUUGCAGGGGUCUAAAACTCATTTCCATTCATAGTAACTATCATTT
	crRNA2	CACCCGUGCAAAAUUGCAGGGGUCUAAAACTTTGAAAGTCATTTCCATTCATAGTAAC
	crRNA3	CACCCGUGCAAAAUUGCAGGGGUCUAAAACTTCCTCCTGTTTGAAAGTCATTTCCATT
Probe	PoRV-P	FAM-CGCACTGGATTTGTATTTCATAAAC-BHQ
	FQ-ssRNA	6-FAM-UUUUUU-BHQ1
RPA primers	F1	5-TAATACGACTCACTATAGGGTTTGACAATGAGTACAGAACCTCCATGTCG-3
	R1	5-TCATCCATACATACATTATCTATAAAATCA-3
	F2	5-TAATACGACTCACTATAGGGAGTAAAGTAGTACCAAGTAATCCAAAATCA-3
	R2	5-AGTAAAGTAGTACCAAGTAATCCAAAATCA-3
qPCR primers	PoRV-VP6-F	GAATTTACAGAATAGGCGACA
	PoRV-VP6-R	AGTTCCCATCAAGTTATCATG

### 2.3 Expression and purification of EsCas13d

The pET28a-MH6-EsCas13d plasmid (Addgene, plasmid #108303, MA, USA) was transformed into *Escherichia coli* BL21 (DE3). The EsCas13d protein was then expressed and purified as previously described (Kushawah et al., 2020). To confirm the presence of the purified EsCas13d protein, we performed SDS-PAGE analysis. For this, we employed a mouse monoclonal anti-6 × His tag antibody (Sangon, Shanghai, China) as the primary antibody, and an HRP-conjugated rabbit anti-mouse IgG (Sangon, Shanghai, China) as the secondary antibody.

### 2.4 *In vitro* transcription and purification of crRNAs

The PoRV VP6 gene was selected as the target for crRNA design using the CRISPR-DT tool (Zhu and Liang, 2019). The DNA template (gDNAs) is the reverse complement of the target crRNA sequence, synthesized with an additional T7 RNA polymerase promoter to facilitate T7 transcription, as detailed in Table 1. These gDNAs were synthesized by Sangon Biotech Co., Ltd. (Shanghai, China). Using the T7 Quick High Yield RNA Transcription Kit (Beyotime, Shanghai, China), the gDNAs were transcribed into crRNA and purified using the Spin Column RNA Cleanup&Concentration Kit (Sangon, Shanghai, China). The concentration of crRNA was measured using the RNA Quick Quantification Kit (Thermo Scientific, MA, USA) and stored at −80°C for future experiments.

### 2.5 Verification of EsCas13d RNase activity

Based on previous studies (Li et al., 2020; You et al., 2024a), a buffer solution for CRISPR/EsCas13d trans-cleavage experiments was prepared, consisting of 40 mM HEPES (pH 7.1), 100 mM KCl, 10 mM MgCl_2_, and 10% glycerol, referred to as Buffer 1. In this system, MgCl_2_ primarily serves as a catalyst and plays a role in stabilizing the reaction environment. The total volume of the EsCas13d reaction system was 20 μL, comprising 10 μL Buffer 1, 500 nM EsCas13d, 200 nM crRNA, 10^6^ pM target RNA, 1 μL RNase inhibitor (2.5 U/μL), and 1 μM FQ-ssDNA reporter construct, with the remaining volume made up with nuclease-free water. The mixture was incubated at 37°C for 30 min in a QuantStudio™ 1PLUS real-time PCR system (Thermo Scientific, MA, USA), with fluorescence readings (FAM channl) collected every minute. After the reaction, the system was exposed to 365 nm UV light for visual inspection.

### 2.6 Recombinase polymerase amplification assay

Primers targeting the PoRV VP6 gene were designed using Primer Premier 5.0 (Premier Biosoft, CA, USA). RPA amplification was performed using the RPA Nucleic Acid Amplification Kit (Warbio, Nanjing, China) according to the manufacturer’s instructions. The total reaction volume was 50 μL, containing 2 μL each of RPA-F and RPA-R primers (10 μM), 25 μL Buffer A, 13.5 μL ultrapure water, 2.5 μL Buffer B, and 5 μL template cDNA. Amplification was carried out at a constant temperature of 37°C for 30 min, and the products were analyzed using agarose gel electrophoresis.

### 2.7 RPA-EsCas13d-based assay

The complete RPA-EsCas13d fluorescence detection system consisted of the following components: 500 nM purified EsCas13d, 200 nM crRNA, 1 μL T7 RNA polymerase (2.5 U/μL), rNTP mix (1 mM), 1 μL RNase inhibitor (2.5 U/μL), 1 μL template (amplified RPA product), 1 μM FQ-ssRNA reporter gene, 10 μL reaction buffer (Buffer 1), and nuclease-free water to bring the total volume to 20 μL. The mixture was incubated at 37°C for 30 min in a QuantStudio™ 1 PLUS real-time PCR system (Thermo Scientific, MA, USA), with fluorescence measurements (FAM) taken every minute. For the negative control (NC), 2 μL of nuclease-free water was used in place of the template.

### 2.8 One-pot RPA-EsCas13d (OP-RPA-EsCas13d) assay

**Figure 1:**
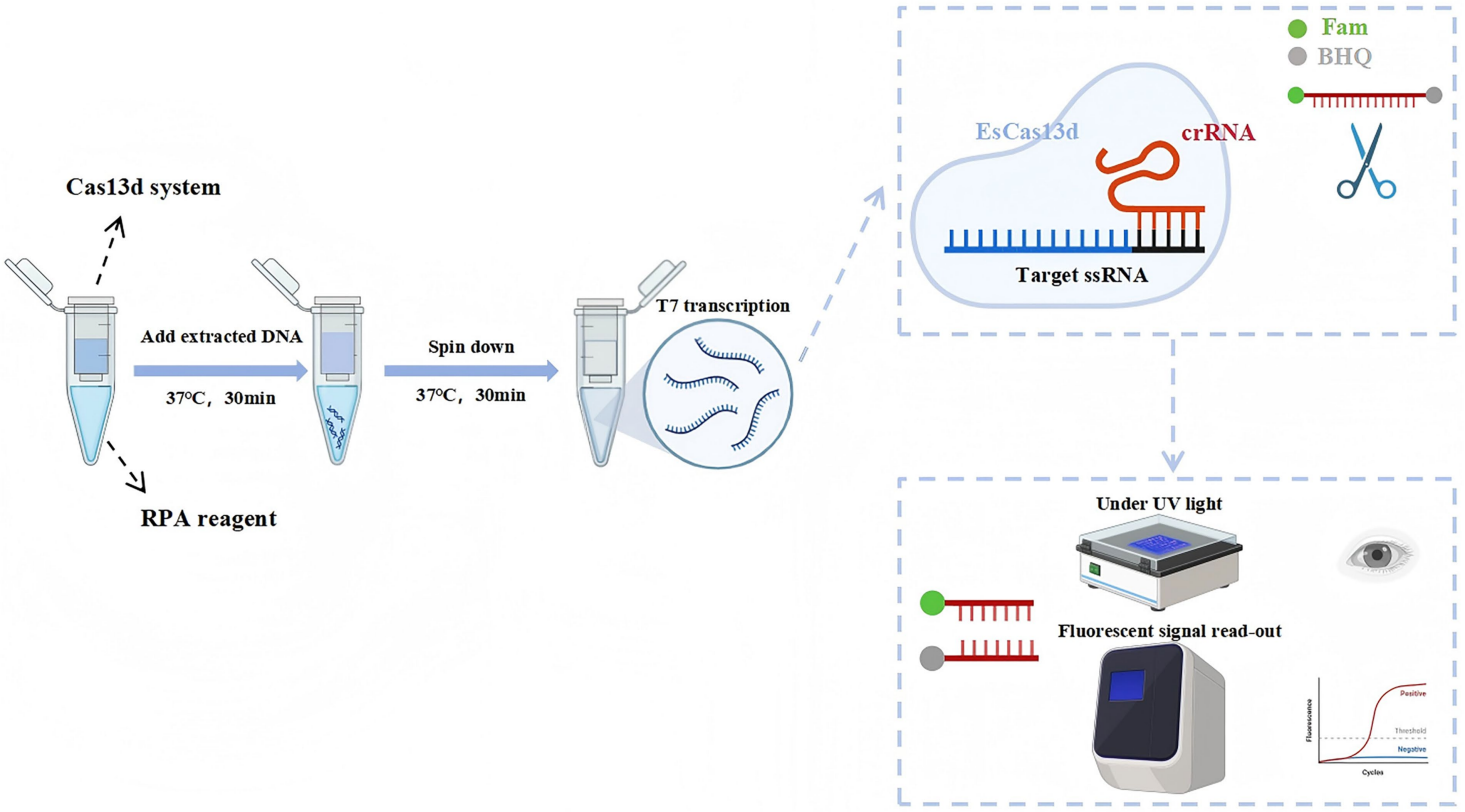
Schematic of the RPA-CRISPR/EsCas13d detection platform. DNA is amplified using RPA and subsequently transcribed with T7 RNA polymerase. The transcribed RNA is then specifically bound and cleaved by the EsCas13d/crRNA complex, which activates the collateral cleavage activity of EsCas13d. The final detection results can be visualized through fluorescence and UV.

The OP-RPA-EsCas13d detection platform integrates RPA amplification and Cas13d detection into a single reaction tube. The detection workflow is as shown by previous researchers (You et al., 2024b). RPA was performed at the bottom of the outer tube, with a 10 μL RPA reaction system consisting of 5 μL RPA Buffer A, 0.5 μL RPA Buffer B, 2 μM forward and reverse primers, 1 mM rNTP mix, 1 μL template cDNA (4 × 10^6^ copies/μL), and ultrapure water to complete the volume. In the inner tube, 20 μL of the EsCas13d reaction system was added, including 500 nM purified EsCas13d, 200 nM crRNA, 1 μM FQ-ssDNA reporter gene, 1 μL T7 RNA polymerase (2.5 U/μL), 1 μL RNase inhibitor (2.5 U/μL), and 10 μL Buffer 1. The tube was then sealed and placed in a thermal cycler or an isothermal module to ensure temperature stability. The RPA amplification was carried out at 37°C for 30 min. After amplification, the tube was briefly centrifuged to combine the EsCas13d system in the inner tube with the RPA product. The combined mixture was incubated at 37°C for an additional 30 min in the QuantStudio™ 1 PLUS real-time PCR system (Thermo Scientific, MA, USA), with fluorescence readings (FAM) collected every minute. For the negative control (NC), 2 μL of nuclease-free water was used in place of the template. The detailed flowchart of the detection process is presented in Figure 1.

### 2.9 Optimization of the EsCas13d detection system

The concentrations of ssDNA reporter molecules, EsCas13d, and crRNA are key factors affecting the efficiency of the CRISPR/EsCas13d reaction. The OP-RPA-EsCas13d method was optimized to enhance its performance. Additionally, the concentration of the ssRNA reporter gene was optimized across six levels (125, 250, 500, 750, 1,000, and 1,500 nM) to balance reagent cost and reaction performance. To address spatial effects and competitive interactions within the system, the ratio of Cas13d to crRNA was adjusted, using 200 nM crRNA and varying Cas13d concentrations from 100 to 600 nM. All experiments used nuclease-free water as a negative control.

### 2.10 Sensitivity experiment of the OP-RPA-EsCas13d detection system

To determine the concentration of the PoRV standard, the copy number was calculated using the corresponding formula. The plasmid was then serially diluted in 10-fold gradients using ddH_2_O to obtain test samples with copy numbers ranging from 10^7^ to 10^1^ copies/μL. Following the procedure outlined in step 2.8 and using the optimal reaction system, the test samples were analyzed using the OP-CRISPR/Cas13d detection system. After completion, the samples were observed under UV light to determine the detection limit of the OP-CRISPR/Cas13d system.

### 2.11 Identification of RV in clinical samples

The performance of the OP-RPA-EsCas13d detection method was evaluated by testing 62 clinical sample. These samples included 40 fecal samples from diarrheic piglets and 22 intestinal tissue samples from pigs that died due to diarrhea. All samples were collected between April and October 2024 from vegetation-rich mountainous regions in southwestern China. For fecal samples, an appropriate amount of physiological saline is added, mixed thoroughly, and then centrifuged to collect the supernatant. For intestinal tissue, the tissue is homogenized in physiological saline, followed by centrifugation to obtain the supernatant. This process prepares the fecal and intestinal tissue samples. The OP-RPA-CRISPR/EsCas13d platform and RT-qPCR was used for detection. RT-qPCR primers and probes are shown in Table 1. The reaction mixtures, performed in three technical replicates, consisted of the following components: 10 μL of TaqMan™ Universal PCR Master Mix (Thermo Scientific, MA, USA), 200 nM of forward and reverse primers, 1 μL of probe, 2 μL of template cDNA, and the necessary amount of ddH2O to achieve a final reaction volume of 20 μL. The cycling conditions included an initial denaturation step at 95°C for 5 min, followed by 40 cycles of denaturation at 95°C for 15 s, annealing at 60°C for 30 s, and extension at 72°C for 30 s. In the RT-qPCR, a Ct value below 36 is considered positive.

### 2.12 Statistical analysis

Graphs were generated using GraphPad Prism 9.0 (GraphPad Software, CA, USA), and all data were presented as the mean ± standard deviation (SD) from three independent experiments. Statistical analyses were performed using SPSS 20.0 (IBM, Chicago, IL, USA). An unpaired t-test was used to compare data between two groups, while one-way analysis of variance (ANOVA) followed by Tukey’s *post hoc* test was applied for comparisons among multiple groups. A *p*-value < 0.05 was considered statistically significant.

## 3 Results

### 3.1 Construction platform based on the working principles of RPA CRISPR/EsCas13d

**Figure 2:**
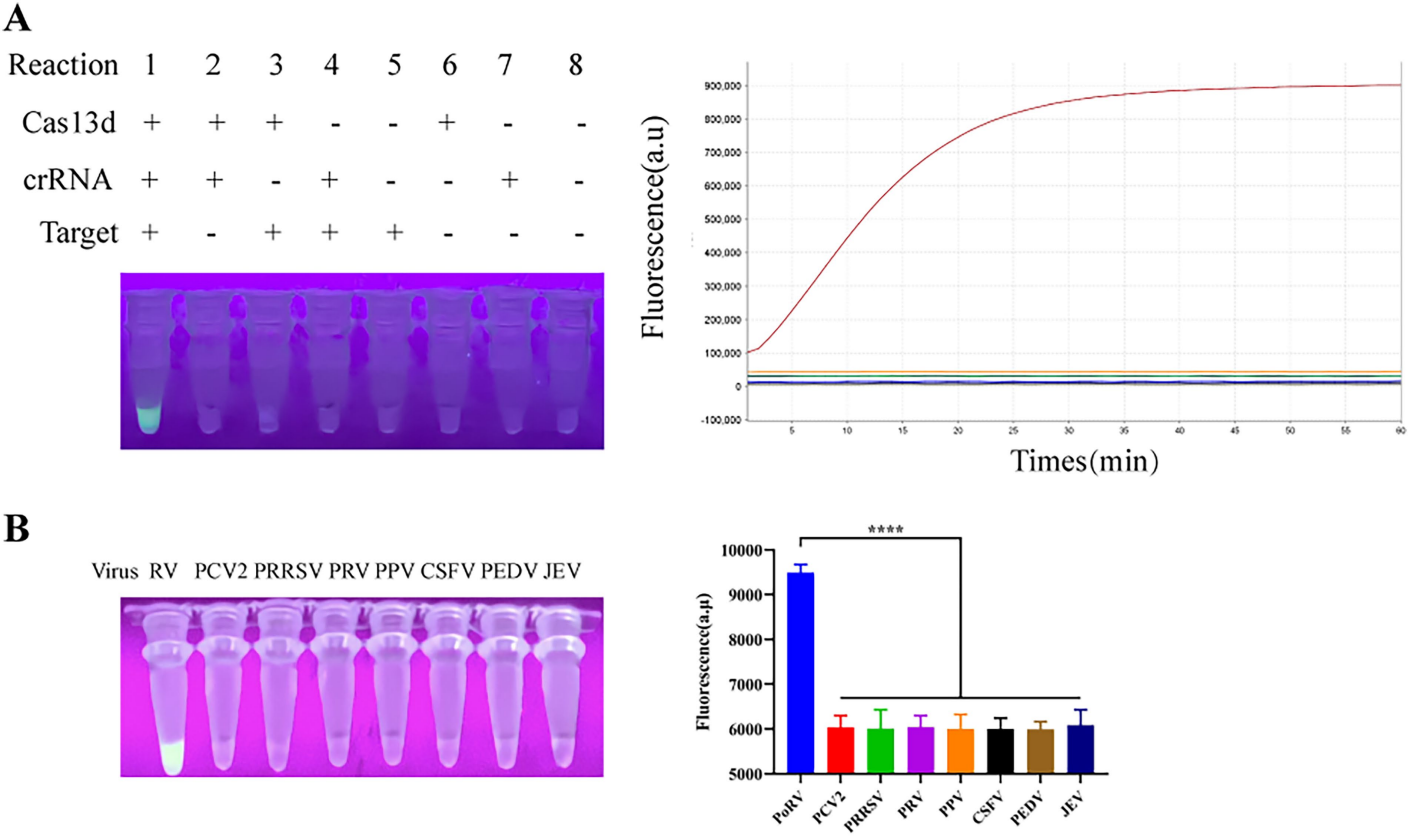
Schematic of the CRISPR/EsCas13d detection platform. **(A)** The cleavage activity of EsCas13d was assessed using Porcine Rotavirus (P0RV)-VP6 as a target, measured by real-time fluorescence and UV light at the reaction endpoint (*n* = 3 replicates; error bars represent SD, ****p < 0.0001). **(B)** The specificity of the CRISPR/EsCas13d detection system.

We first developed a detection system using EsCas13d for the identification of PoRV. The EsCas13d protein was prepared according to previous methods. To demonstrate the cleavage activity of EsCas13d, eight reaction systems were selected for evaluation based on prior articles (Huang et al., 2023). The eight reaction systems each lack one to three essential components of CRISPR/EsCas13d. The results showed that significant fluorescence was observed only in Reaction 1, which included the target gene, FQ-ssRNA reporter protein, crRNA, and EsCas13d protein. This fluorescence could be detected using a quantitative fluorescence PCR instrument or observed with the naked eye under 365 nm UV light (Figure 2A). These results indicated that the purified EsCas13d protein we expressed had RNA cleavage activity. To further assess the specificity of the EsCas13d detection system, we tested multiple viruses, including PoRV, PCV2, PRRSV, PRV, PPV, CSFV, PEDV, and JEV. The results indicated that a strong fluorescence signal was produced only in the presence of PoRV, demonstrating its high specificity (Figure 2B).

### 3.2 Evaluation of optimization effects for the EsCas13d detection system

**Figure 3:**
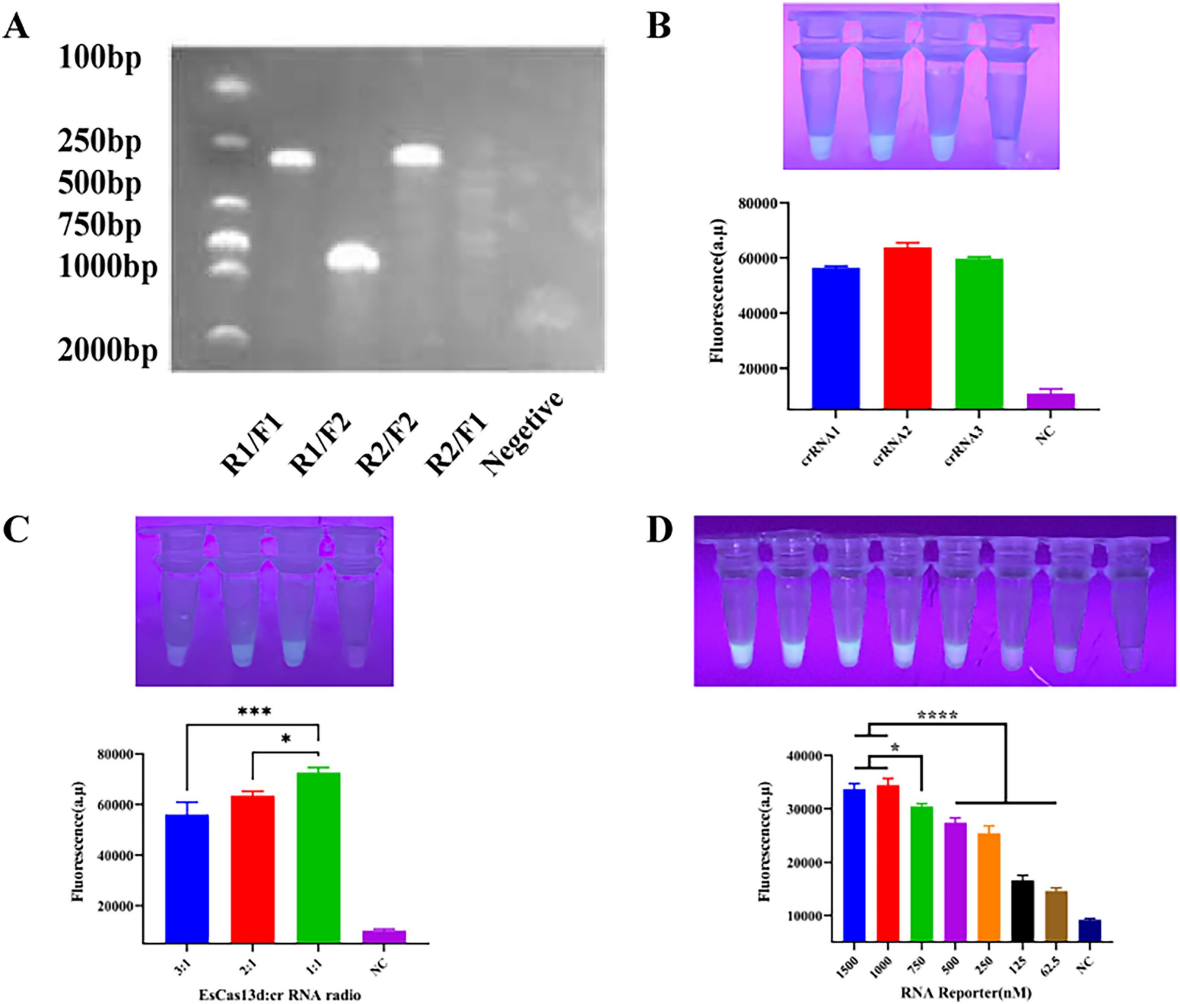
Optimization of CRISPR/EsCas13d. **(A)** Screening for the best RPA primer. The two pairs of RPA primers, R1/F1 and R2/F2, were randomly combined. **(B)** Screening for the best crRNA. **(C)** Optimization of the ratio of EsCas13d to crRNA. EsCas13d: The ratios of crRNA were 600 nM: 200 nM, 400 nM: 200 nM, and 200 nM: 200 nM. **(D)** Optimization of the concentration of the FAS-ssDNA reporter gene.

To enhance the sensitivity of the entire RPA-CRISPR/EsCas13d system, we conducted screening and optimization of various key components and reaction conditions. Firstly, two pairs of PRA primers were designed based on the conserved region of the PoRV VP6 gene. The primers were tested in different combinations using RPA and gel electrophoresis to determine the optimal pair. Results showed that all primer combinations generated target bands of the expected size, while no bands were observed in the negative control due to the absence of RPA primers (Figure 3A). The R2/F2 primer combination exhibited relatively high amplification efficiency and minimal nonspecific amplification, with a product size of approximately 250 bp. Therefore, it was selected for subsequent experiments. For the detection of Porcine Rotavirus (PoRV), we designed three candidate crRNAs based on the conserved region of the PoRV VP6 gene. We then combined the RPA method with the EsCas13d cleavage system. Initially, nucleic acids were pre-amplified using RPA, and the resulting products were used as templates for detection by EsCas13d. Fluorescence signals were measured using a real-time PCR instrument. The results demonstrated that all three crRNAs effectively activated the trans-cleavage activity of EsCas13d, resulting in strong fluorescence signals (Figure 3B). Among them, crRNA2 exhibited the highest fluorescence intensity and cleavage efficiency, making it the preferred choice for further studies. To optimize the CRISPR/ESCas13d detection system, we systematically evaluated its components. Subsequently, we adjusted the ratio of Cas13d to crRNA, with a 1:1 ratio yielding the optimal fluorescence signal (Figure 3C). Finally, we optimized the concentration of the ssRNA reporter gene to balance reagent cost and performance (Figure 3D). The final reaction system for EsCas13d consisted of 10 μL buffer, 1 μM FQ-ssRNA, 200 nM crRNA, 200 nM EsCas13d, 10^−6^ pM target RNA, and 1 μL RNase inhibitor (2.5 U/μL), with the remaining components diluted in nuclease-free water.

### 3.3 Sensitivity evaluation of the RPA-CRISPR/EsCas13d

**Figure 4:**
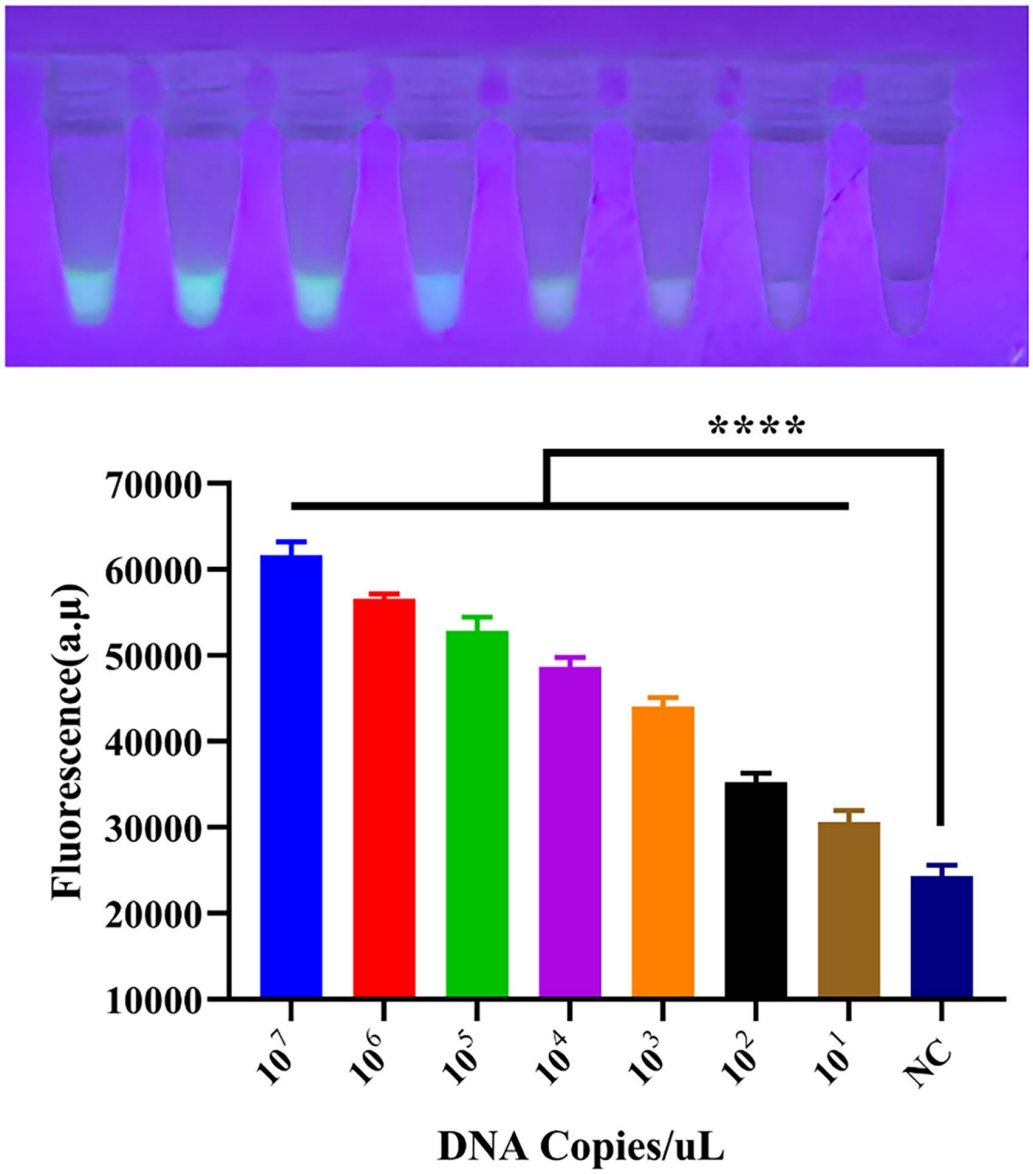
Sensitivity of the CRISPR/EsCas13d detection system. A tenfold dilution series of the PoRV standard was prepared from 10^7^ to 10^1^, and then analyzed using the CRISPR/EsCas13d detection platform.

Using the optimal reaction system determined in section 3.3, the sensitivity of the detection system was tested. We performed a tenfold serial dilution of the standard plasmid, with a dilution range from 1 × 10^7^ to 1 × 10^1^, as shown in Figure 4. As the template concentration decreased, the detected fluorescence intensity also decreased; however, even at as low as 10 copies, a faint fluorescence was still emitted, classified as weak positive. This indicates that the detection limit of the CRISPR/EsCas13d detection system is 10 copies/μL.

### 3.4 Detection of PoRV from clinical samples with the RPA-CRISPR/EsCas13d portable platform and RT-qPCR

**Figure 5:**
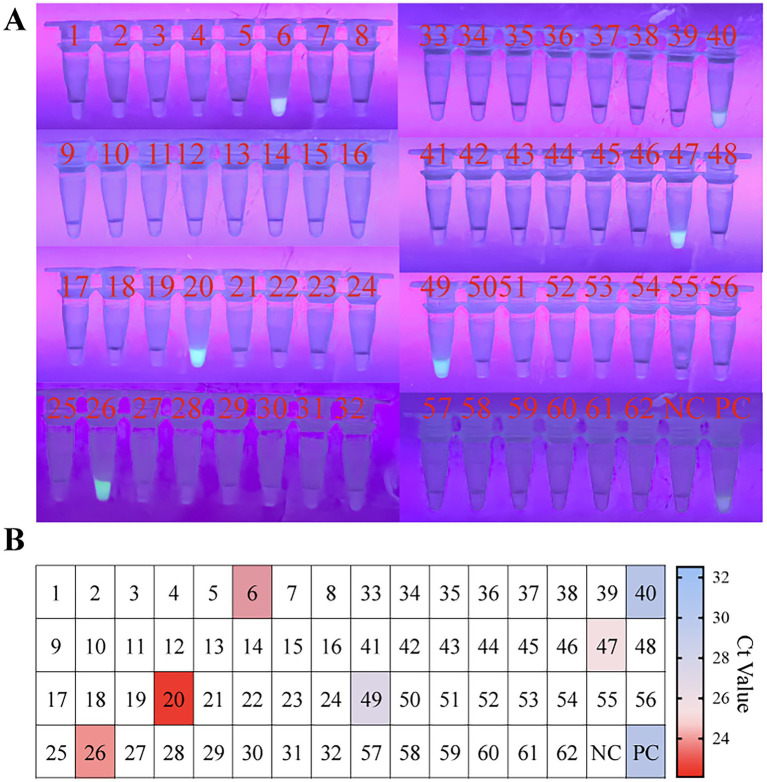
Evaluating the RPA-CRISPR/EsCas13d detection platform in the clinical setting. A total of 40 fecal samples and 22 clinical samples of intestinal tissue were analyzed using RT-qPCR and OP-RPA-EsCas13d. In panels **(A,B)**, samples 1–40 represented 40 fecal samples, while samples 41–62 represented 22 intestinal tissue samples. The colors range from blue to red, representing different Ct values of RT-qPCR, while white indicates a negative result.

To evaluate the feasibility of the RPA-EsCas13d detection system for identifying PoRV in clinical specimens, we analyzed 62 diarrhea samples from the Sichuan region of China. The samples included 40 fecal specimens and 20 intestinal tissue samples. Detection was performed using visible fluorescence, with a reaction time set to 30 min. The results showed that 5 out of the 62 clinical samples (coded as 6, 20, 26, 47, and 50) exhibited strong fluorescence signals (Figure 5), and the results were consistent with those obtained from RT-qPCR (Figure 5). The positive detection rate was 8.06% (5/62), confirming the consistency and reliability of the CRISPR/EsCas13d and RT-qPCR methods. For detailed information regarding RT-qPCR, please refer to Supplementary file 2.

## 4 Discussion

In the past, PoRV was considered a major cause of diarrhea in infants and young animals. According to the World Health Organization, RV causes approximately 450,000 deaths annually, with 90% occurring in developing countries in Asia and Africa (Sadiq and Khan, 2023). PoRV is widely distributed worldwide, and its zoonotic nature has led to significant economic losses in the swine industry. Currently, vaccination remains the primary strategy for PoRV prevention and control. However, in resource-limited regions and small to medium-sized pig farms, vaccine coverage remains insufficient, increasing the risk of outbreaks (Bibera et al., 2020). Therefore, efficient, accurate, and user-friendly diagnostic methods are crucial for PoRV prevention and control.

In this study, we successfully developed a visualized one-pot RPA-EsCas13d detection system, which enables fluorescence-based visualization under portable blue and UV light. The standalone CRISPR/Cas13d detection system struggles to detect pathogenic signals at low target concentrations. However, by integrating RPA technology, we enhanced CRISPR-mediated cleavage signals while combining the specificity of RPA amplification with crRNA sequence recognition. This synergy significantly improved the sensitivity and specificity of Cas13d-based detection. The combination of these two technologies allowed the OP-RPA-EsCas13d detection system to achieve a detection limit as low as 10 copies in fluorescence-based assays, demonstrating superior performance compared to most existing detection methods (Hou et al., 2023; Hu et al., 2016; Jia et al., 2019; Shi et al., 2021).

Rapid and accurate nucleic acid detection is crucial for virus diagnostics, genetically modified organism identification, genotyping, and disease diagnosis. Conventional molecular diagnostic methods like PCR, hybridization, and isothermal amplification often face limitations in specificity, sensitivity, cost, or complexity. Recently, the collateral effects of CRISPR-Cas systems, which offer highly specific target recognition, have been widely applied for nucleic acid detection. CRISPR-Cas12-based systems such as DETECTR and HOLMES, as well as Cas13-based systems like SHERLOCK, have demonstrated excellent specificity and sensitivity for rapid DNA and RNA detection (Jex et al., 2025; Kellner et al., 2019; Khmeleva et al., 2024; Kostyusheva et al., 2022; Zhuang et al., 2024). CRISPR-Cas12 has been used for diagnosing various swine pathogens, including JEV, PRRSV, ASFV, and *Mycoplasma pneumoniae* (Liu et al., 2021; Wu et al., 2020; You et al., 2024b). Chen and Huang et al. developed detection platforms for rotavirus by combining NASBA and RPA amplification techniques with CRISPR/Cas12a (Chen et al., 2024; Huang et al., 2024). Both platforms can detect positive templates with as low as one copy in as little as 60–70 min. However, the detection platforms constructed by both methods have limitations, as nucleic acid amplification and CRISPR secondary amplification are performed in two separate steps. In contrast, our proposed one-step strategy simplifies the operational process and, more importantly, reduces the risk of aerosol contamination. In addition, CRISPR/Cas12 faces greater challenges than CRISPR/Cas13 in terms of protein activity and sgDNA design. Wang et al. also indicated that in single-tube CRISPR reactions, Cas13 outperforms Cas12 for DNA target detection, which seems to be related to the additional T7 transcription step delaying the activation of Cas13 (Wang et al., 2024). However, this study did not find evidence that Cas13 exhibits superior detection sensitivity compared to Cas12. Nevertheless, the design advantages and compact size of Cas13 are sufficient to make it a superior choice for detection systems compared to Cas12.

Undoubtedly, our study has certain limitations in some aspects. Although the one-pot strategy we employed significantly reduced aerosol contamination, we still need to first reverse transcribe the PoRV genome into cDNA and then add the nucleic acids to the CRISPR/EsCas13d reaction system for amplification and visualization, which carries the risk of contamination. Combining RT with PRA in a single tube to reduce contamination risks and improve operability is a strategy we plan to consider in the future. Moreover, common pathogens that cause diarrhea in piglets, such as PED, PDCoV, and TGEV, often present similar clinical symptoms that are difficult to distinguish visually. Future research should extend the RPA-EsCas13d platform to a multi-pathogen system to differentiate pathogens with similar symptoms, thereby providing more comprehensive diagnostic information for clinical use. Recent research by Ackerman’s team offers a new perspective, where they developed a digital detection technology called CARMEN. Using the CARMEN-Cas13 combination, they can simultaneously distinguish several human-associated viruses, overcoming the limitation of the Cas13d system, which is designed for single-virus analysis (Ackerman et al., 2020). Therefore, our future work aims to overcome these limitations and develop an integrated and multiplexed platform for reverse transcription, amplification, and Cas detection.

## 5 Conclusion

In summary, we have developed a convenient, rapid, and sensitive one-pot PRA-EsCas13d detection system. Our platform requires only one isothermal module, and the results can be directly read with the naked eye. This study enhances the diversity of PoRV detection methods and provides a fast, sensitive, and convenient diagnostic platform for PoRV. It offers an important tool for public health surveillance and disease prevention strategies related to PoRV. In the future, we plan to modify and balance the primers and buffer system within this platform to achieve greater diversity and stability in detection capabilities.

## Data Availability

The original contributions presented in the study are included in the article/Supplementary material, further inquiries can be directed to the corresponding authors.
