# Supplementary material for: Rapid, sensitive, and visual detection of porcine rotavirus with RPA-CRISPR/Cas13
Source: Front Microbiol. 2025 Aug 20;16:1617955. doi: 10.3389/fmicb.2025.1617955 (PMC12405341; doi:10.3389/fmicb.2025.1617955)
Supplement: Supplementary file 1 [file Data_Sheet_1.docx]

**>JQ343834.1 Porcine rotavirus strain PoRV-1/CBNU1 VP6 gene, the conserved region** GGCTTTAAAACGAAGTCTTCGACATGGAGGTTCTGTACTCATTGTCAAACTTTGAGGATGCTAGAGATAAAATTGTTGAAGGTACATTATATTCAAATGTAAGTGATTTGATTCAACAATTTAATCAAATGATAGTTACTATGAATGGAAATGATTTTCAAACGGGAGGAATAGGAAATTTGCCAATCAGGAATTGGACTTTTGATTTTGGGTTACTTGGTACTACTTTACTTAATCTAGATGCAAATTATGTTGAGAATGCTAGAACTACTATTGAATACTTTATTGATTTTATAGATAATGTATGTATGGATGAAATGGCTAGAGAATCACAACGAAATGGGATAGCACCACAATCTGAAGCACTGAGAAAGCTGTCGGGTATCAAATTTAAGAGAATTAATTTTGATAATTCATCTGATTATATTGAGAATTGGAATTTACAGAATAGGCGACAGCGCACTGGATTTGTATTTCATAAACCAAATATACTTCCATATTCAGCATCATTCACTCTAAACCGATCACAGCCAGCTCATGATAACTTGATGGGAACTATGTGGATTAACGCTGGATCAGAAATTCAAGTAGCTGGATTTGATTATTCATGTGCTTTTAATGCACCAGCAAACATTCAGCAGTTTGAGCATGTTGTACCATTAAGACGTGCCCTCACAACAGCTACAATTACTCTACTACCAGATGCTGAAAGATTCAGTTTTCCGAGAGTAATTAACTCAGCTGATGGTACTACCACATGGTATTTCAATCCAGTCATTCTAAGACCAAGTAATGTAGAAGTTGAGTTTTTATTAAATGGACAGACAATTAATACATATCAAGCACGATTTGGAACTATTGTAGCTAGAAATTTTGACACCATTCGCTTATCATTTCAATTAGTACGTCCACCGAACATGACACCAGCAGTTGCAAACCTATTTCCGCAAGCACCACCATTTATATTTCATGCTACAGTTGGACTTACGATGCGCATTGAATCTGCAGTTTGTGAATCTGTGCTTGCGGATGCTTCAGAAACTTTATTGGCAAATGTGACCTCGGTACGTCAAGAGTATGCAATACCAGTAGGACCAGTATTTCCACCAGGTATGAATTGGACAGAATTAATTACCAATTATTCACCTTCAAGAGAAGATAACTTGCAACGTGTTTTTACAGTAGCTTCCATTAGAAGCATGTTGATTAAGTGAGGACTAGGCTAACTACCTGGTATCCGATCTTAACCAGCATGTAACTATGTCAAGTCAATCAGACTCTACAAGTAAGGGTATGACTTCATACTCGCTACGTAGAGTAACTGTTTGAATGGTATAGTGAGAGGATGTGACC
